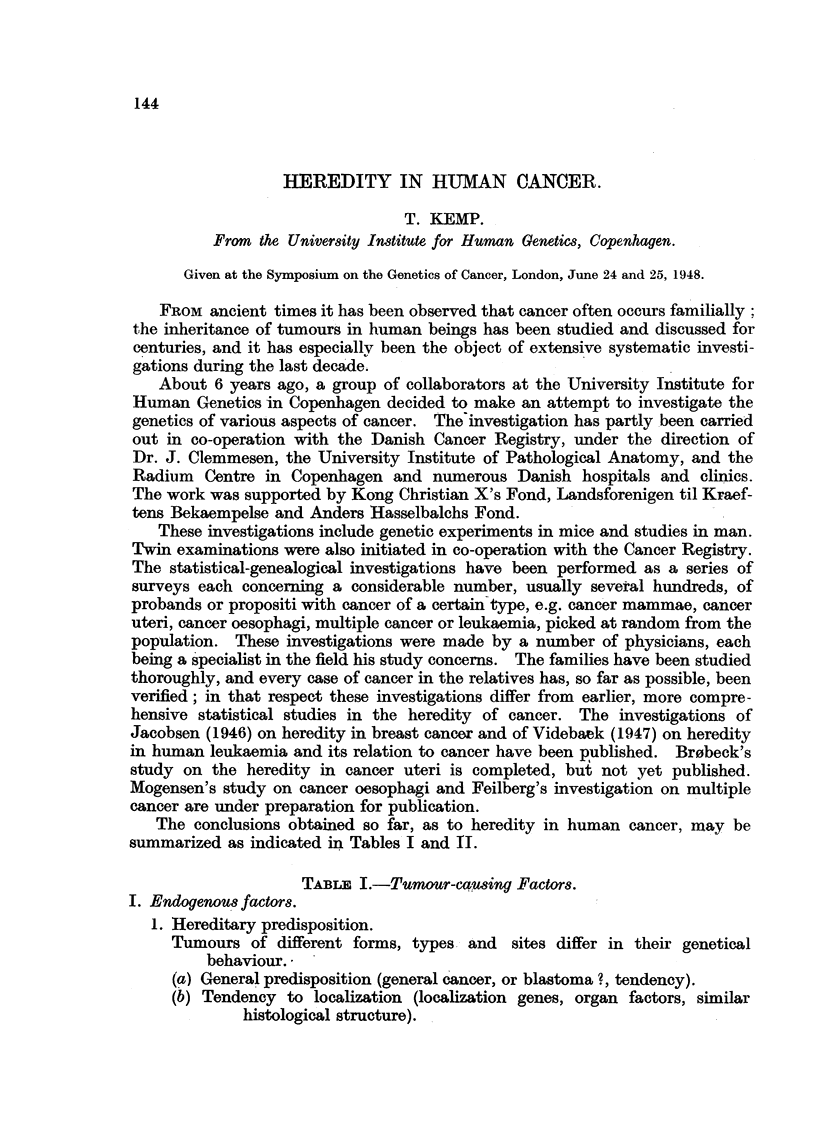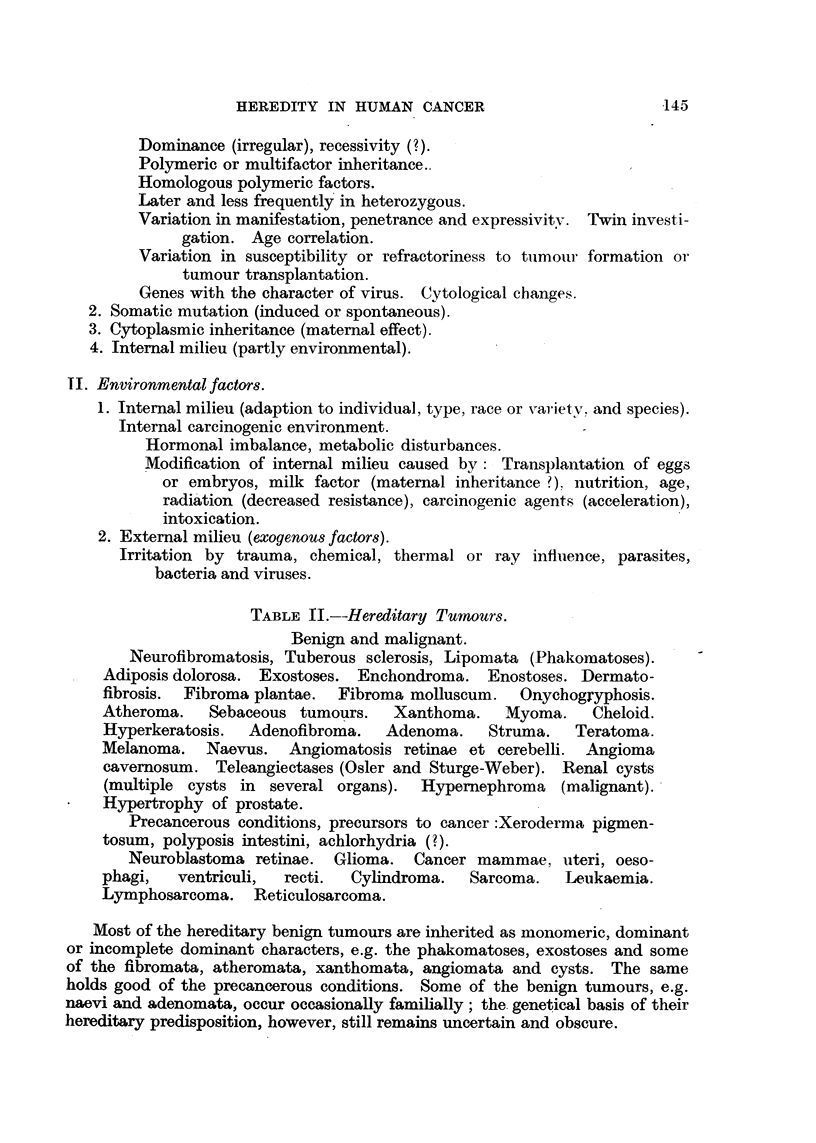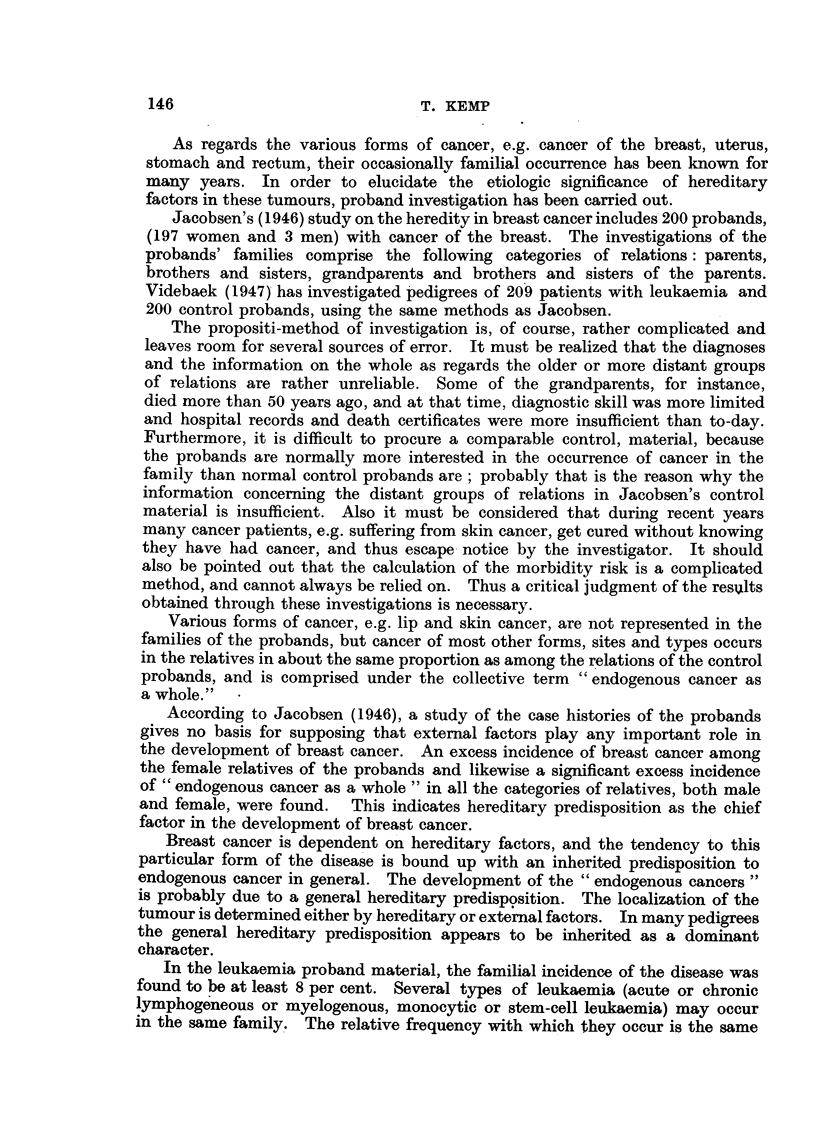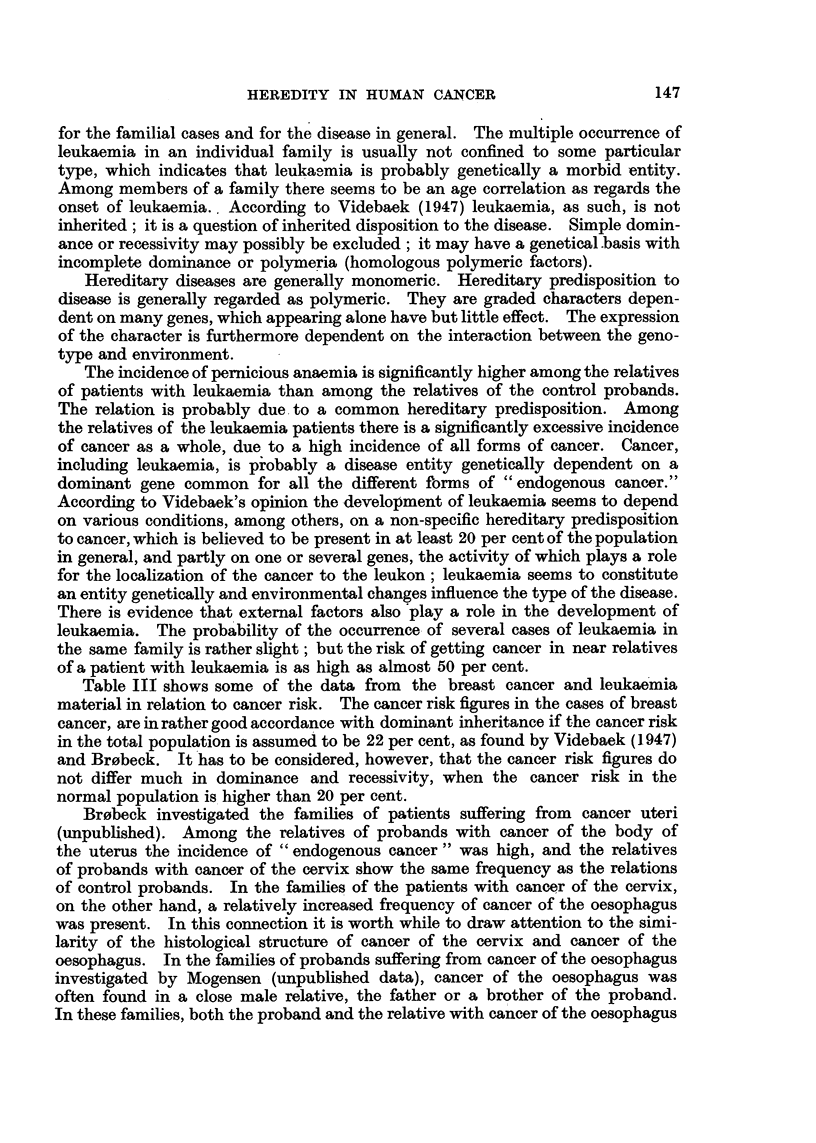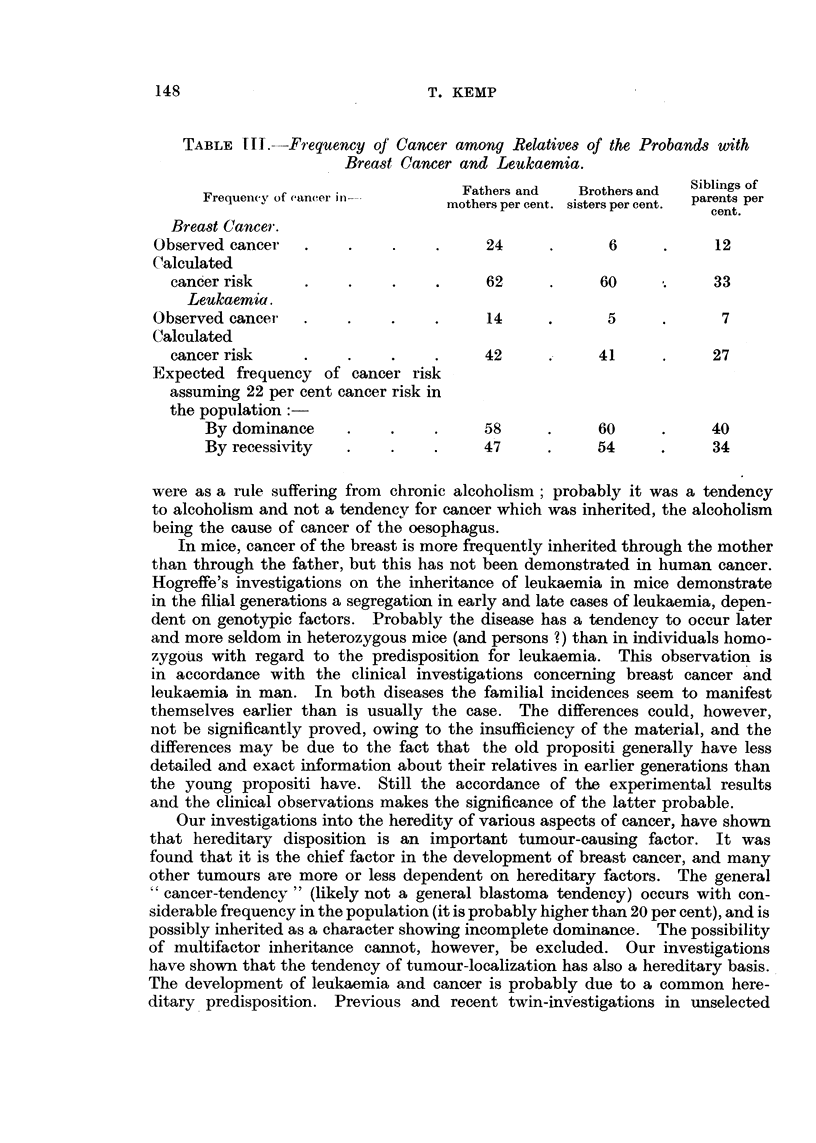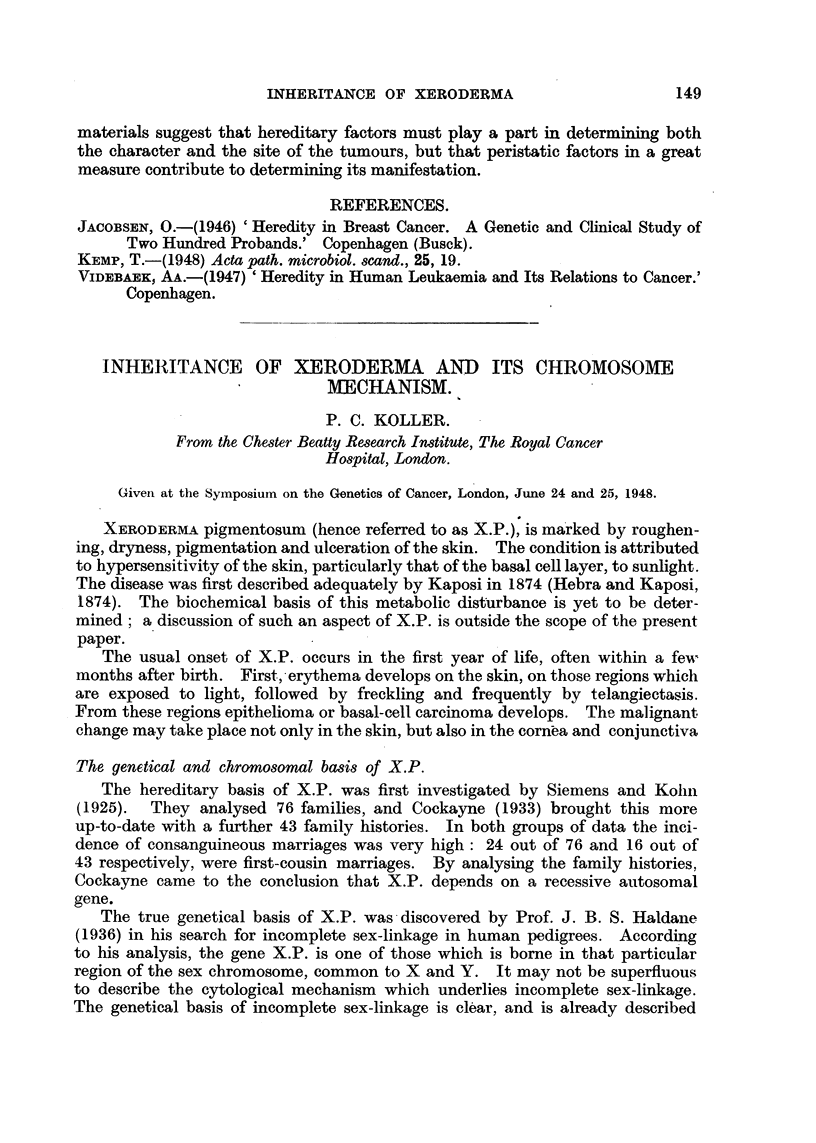# Heredity in Human Cancer

**DOI:** 10.1038/bjc.1948.21

**Published:** 1948-06

**Authors:** T. Kemp


### HEREDITY IN HUMAN CANCER.

T. KEMP.

*From the University Institute for Human Genetics, Copenhagen.*

Given at the Symposium on the Genetics of Cancer, London, June 24 and 25, 1948.

FROM ancient times it has been observed that cancer often occurs familially; the inheritance of tumours in human beings has been studied and discussed for centuries, and it has especially been the object of extensive systematic investigations during the last decade.

About 6 years ago, a group of collaborators at the University Institute for Human Genetics in Copenhagen decided to make an attempt to investigate the genetics of various aspects of cancer. The investigation has partly been carried out in co-operation with the Danish Cancer Registry, under the direction of Dr. J. Clemmesen, the University Institute of Pathological Anatomy, and the Radium Centre in Copenhagen and numerous Danish hospitals and clinics. The work was supported by Kong Christian X's Fond, Landsforenigen til Kraeftens Bekaempelse and Anders Hasselbalchs Fond.

These investigations include genetic experiments in mice and studies in man. Twin examinations were also initiated in co-operation with the Cancer Registry. The statistical-genealogical investigations have been performed as a series of surveys each concerning a considerable number, usually several hundreds, of probands or propositi with cancer of a certain type, e.g. cancer mammae, cancer uteri, cancer oesophagi, multiple cancer or leukaemia, picked at random from the population. These investigations were made by a number of physicians, each being a specialist in the field his study concerns. The families have been studied thoroughly, and every case of cancer in the relatives has, so far as possible, been verified; in that respect these investigations differ from earlier, more comprehensive statistical studies in the heredity of cancer. The investigations of Jacobsen (1946) on heredity in breast cancer and of Videbaek (1947) on heredity in human leukaemia and its relation to cancer have been published. Brøbeck's study on the heredity in cancer uteri is completed, but not yet published. Mogensen's study on cancer oesophagi and Feilberg's investigation on multiple cancer are under preparation for publication.

The conclusions obtained so far, as to heredity in human cancer, may be summarized as indicated in Tables I and II.

TABLE I.—*Tumour-causing Factors.*

I. *Endogenous factors.*

1. Hereditary predisposition.
   Tumours of different forms, types and sites differ in their genetical behaviour.
   (*a*) General predisposition (general cancer, or blastoma ?, tendency).
   (*b*) Tendency to localization (localization genes, organ factors, similar histological structure).

Dominance (irregular), recessivity (?).
Polymeric or multifactor inheritance..
Homologous polymeric factors.
Later and less frequently in heterozygous.
Variation in manifestation, penetrance and expressivity. Twin investigation. Age correlation.
Variation in susceptibility or refractoriness to tumour formation or tumour transplantation.
Genes with the character of virus. Cytological changes.

2. Somatic mutation (induced or spontaneous).
3. Cytoplasmic inheritance (maternal effect).
4. Internal milieu (partly environmental).

II. *Environmental factors.*

1. Internal milieu (adaption to individual, type, race or variety, and species).
Internal carcinogenic environment.
Hormonal imbalance, metabolic disturbances.
Modification of internal milieu caused by: Transplantation of eggs or embryos, milk factor (maternal inheritance ?), nutrition, age, radiation (decreased resistance), carcinogenic agents (acceleration), intoxication.
2. External milieu (*exogenous factors*).
Irritation by trauma, chemical, thermal or ray influence, parasites, bacteria and viruses.

TABLE II.—*Hereditary Tumours.*

Benign and malignant.

Neurofibromatosis, Tuberous sclerosis, Lipomata (Phakomatoses). Adiposis dolorosa. Exostoses. Enchondroma. Enostoses. Dermatofibrosis. Fibroma plantae. Fibroma molluscum. Onychogryphosis. Atheroma. Sebaceous tumours. Xanthoma. Myoma. Cheloid. Hyperkeratosis. Adenofibroma. Adenoma. Struma. Teratoma. Melanoma. Naevus. Angiomatosis retinae et cerebelli. Angioma cavernosum. Teleangiectases (Osler and Sturge-Weber). Renal cysts (multiple cysts in several organs). Hypernephroma (malignant). Hypertrophy of prostate.

Precancerous conditions, precursors to cancer :Xeroderma pigmentosum, polyposis intestini, achlorhydria (?).

Neuroblastoma retinae. Glioma. Cancer mammae, uteri, oesophagi, ventriculi, recti. Cylindroma. Sarcoma. Leukaemia. Lymphosarcoma. Reticulosarcoma.

Most of the hereditary benign tumours are inherited as monomeric, dominant or incomplete dominant characters, e.g. the phakomatoses, exostoses and some of the fibromata, atheromata, xanthomata, angiomata and cysts. The same holds good of the precancerous conditions. Some of the benign tumours, e.g. naevi and adenomata, occur occasionally familially ; the genetical basis of their hereditary predisposition, however, still remains uncertain and obscure.

As regards the various forms of cancer, e.g. cancer of the breast, uterus, stomach and rectum, their occasionally familial occurrence has been known for many years. In order to elucidate the etiologic significance of hereditary factors in these tumours, proband investigation has been carried out.

Jacobsen's (1946) study on the heredity in breast cancer includes 200 probands, (197 women and 3 men) with cancer of the breast. The investigations of the probands' families comprise the following categories of relations: parents, brothers and sisters, grandparents and brothers and sisters of the parents. Videbaek (1947) has investigated pedigrees of 209 patients with leukaemia and 200 control probands, using the same methods as Jacobsen.

The propositi-method of investigation is, of course, rather complicated and leaves room for several sources of error. It must be realized that the diagnoses and the information on the whole as regards the older or more distant groups of relations are rather unreliable. Some of the grandparents, for instance, died more than 50 years ago, and at that time, diagnostic skill was more limited and hospital records and death certificates were more insufficient than to-day. Furthermore, it is difficult to procure a comparable control, material, because the probands are normally more interested in the occurrence of cancer in the family than normal control probands are ; probably that is the reason why the information concerning the distant groups of relations in Jacobsen's control material is insufficient. Also it must be considered that during recent years many cancer patients, e.g. suffering from skin cancer, get cured without knowing they have had cancer, and thus escape notice by the investigator. It should also be pointed out that the calculation of the morbidity risk is a complicated method, and cannot always be relied on. Thus a critical judgment of the results obtained through these investigations is necessary.

Various forms of cancer, e.g. lip and skin cancer, are not represented in the families of the probands, but cancer of most other forms, sites and types occurs in the relatives in about the same proportion as among the relations of the control probands, and is comprised under the collective term "endogenous cancer as a whole."

According to Jacobsen (1946), a study of the case histories of the probands gives no basis for supposing that external factors play any important role in the development of breast cancer. An excess incidence of breast cancer among the female relatives of the probands and likewise a significant excess incidence of "endogenous cancer as a whole" in all the categories of relatives, both male and female, were found. This indicates hereditary predisposition as the chief factor in the development of breast cancer.

Breast cancer is dependent on hereditary factors, and the tendency to this particular form of the disease is bound up with an inherited predisposition to endogenous cancer in general. The development of the "endogenous cancers" is probably due to a general hereditary predisposition. The localization of the tumour is determined either by hereditary or external factors. In many pedigrees the general hereditary predisposition appears to be inherited as a dominant character.

In the leukaemia proband material, the familial incidence of the disease was found to be at least 8 per cent. Several types of leukaemia (acute or chronic lymphogeneous or myelogenous, monocytic or stem-cell leukaemia) may occur in the same family. The relative frequency with which they occur is the same for the familial cases and for the disease in general. The multiple occurrence of leukaemia in an individual family is usually not confined to some particular type, which indicates that leukaemia is probably genetically a morbid entity. Among members of a family there seems to be an age correlation as regards the onset of leukaemia. According to Videbaek (1947) leukaemia, as such, is not inherited ; it is a question of inherited disposition to the disease. Simple dominance or recessivity may possibly be excluded ; it may have a genetical basis with incomplete dominance or polymeria (homologous polymeric factors).

Hereditary diseases are generally monomeric. Hereditary predisposition to disease is generally regarded as polymeric. They are graded characters dependent on many genes, which appearing alone have but little effect. The expression of the character is furthermore dependent on the interaction between the genotype and environment.

The incidence of pernicious anaemia is significantly higher among the relatives of patients with leukaemia than among the relatives of the control probands. The relation is probably due to a common hereditary predisposition. Among the relatives of the leukaemia patients there is a significantly excessive incidence of cancer as a whole, due to a high incidence of all forms of cancer. Cancer, including leukaemia, is probably a disease entity genetically dependent on a dominant gene common for all the different forms of "endogenous cancer." According to Videbaek's opinion the development of leukaemia seems to depend on various conditions, among others, on a non-specific hereditary predisposition to cancer, which is believed to be present in at least 20 per cent of the population in general, and partly on one or several genes, the activity of which plays a role for the localization of the cancer to the leukon ; leukaemia seems to constitute an entity genetically and environmental changes influence the type of the disease. There is evidence that external factors also play a role in the development of leukaemia. The probability of the occurrence of several cases of leukaemia in the same family is rather slight ; but the risk of getting cancer in near relatives of a patient with leukaemia is as high as almost 50 per cent.

Table III shows some of the data from the breast cancer and leukaemia material in relation to cancer risk. The cancer risk figures in the cases of breast cancer, are in rather good accordance with dominant inheritance if the cancer risk in the total population is assumed to be 22 per cent, as found by Videbaek (1947) and Brøbeck. It has to be considered, however, that the cancer risk figures do not differ much in dominance and recessivity, when the cancer risk in the normal population is higher than 20 per cent.

Brøbeck investigated the families of patients suffering from cancer uteri (unpublished). Among the relatives of probands with cancer of the body of the uterus the incidence of "endogenous cancer" was high, and the relatives of probands with cancer of the cervix show the same frequency as the relations of control probands. In the families of the patients with cancer of the cervix, on the other hand, a relatively increased frequency of cancer of the oesophagus was present. In this connection it is worth while to draw attention to the similarity of the histological structure of cancer of the cervix and cancer of the oesophagus. In the families of probands suffering from cancer of the oesophagus investigated by Mogensen (unpublished data), cancer of the oesophagus was often found in a close male relative, the father or a brother of the proband. In these families, both the proband and the relative with cancer of the oesophagus

TABLE III.—*Frequency of Cancer among Relatives of the Probands with Breast Cancer and Leukaemia.*

| Frequency of cancer in— | Fathers and mothers per cent. | Brothers and sisters per cent. | Siblings of parents per cent. |
|---|---|---|---|
| *Breast Cancer.* | | | |
| Observed cancer | 24 | 6 | 12 |
| Calculated cancer risk | 62 | 60 | 33 |
| *Leukaemia.* | | | |
| Observed cancer | 14 | 5 | 7 |
| Calculated cancer risk | 42 | 41 | 27 |
| Expected frequency of cancer risk assuming 22 per cent cancer risk in the population :— | | | |
| By dominance | 58 | 60 | 40 |
| By recessivity | 47 | 54 | 34 |

were as a rule suffering from chronic alcoholism ; probably it was a tendency to alcoholism and not a tendency for cancer which was inherited, the alcoholism being the cause of cancer of the oesophagus.

In mice, cancer of the breast is more frequently inherited through the mother than through the father, but this has not been demonstrated in human cancer. Hogreffe's investigations on the inheritance of leukaemia in mice demonstrate in the filial generations a segregation in early and late cases of leukaemia, dependent on genotypic factors. Probably the disease has a tendency to occur later and more seldom in heterozygous mice (and persons ?) than in individuals homozygous with regard to the predisposition for leukaemia. This observation is in accordance with the clinical investigations concerning breast cancer and leukaemia in man. In both diseases the familial incidences seem to manifest themselves earlier than is usually the case. The differences could, however, not be significantly proved, owing to the insufficiency of the material, and the differences may be due to the fact that the old propositi generally have less detailed and exact information about their relatives in earlier generations than the young propositi have. Still the accordance of the experimental results and the clinical observations makes the significance of the latter probable.

Our investigations into the heredity of various aspects of cancer, have shown that hereditary disposition is an important tumour-causing factor. It was found that it is the chief factor in the development of breast cancer, and many other tumours are more or less dependent on hereditary factors. The general "cancer-tendency" (likely not a general blastoma tendency) occurs with considerable frequency in the population (it is probably higher than 20 per cent), and is possibly inherited as a character showing incomplete dominance. The possibility of multifactor inheritance cannot, however, be excluded. Our investigations have shown that the tendency of tumour-localization has also a hereditary basis. The development of leukaemia and cancer is probably due to a common hereditary predisposition. Previous and recent twin-investigations in unselected materials suggest that hereditary factors must play a part in determining both the character and the site of the tumours, but that peristatic factors in a great measure contribute to determining its manifestation.

### REFERENCES.

JACOBSEN, O.—(1946) 'Heredity in Breast Cancer. A Genetic and Clinical Study of Two Hundred Probands.' Copenhagen (Busck).

KEMP, T.—(1948) *Acta path. microbiol. scand.*, **25**, 19.

VIDEBAEK, AA.—(1947) 'Heredity in Human Leukaemia and Its Relations to Cancer.' Copenhagen.

---